# Nuclear medicine practices during the COVID-19 pandemic—review of some recently published protocols

**DOI:** 10.1186/s43055-020-00349-1

**Published:** 2020-11-16

**Authors:** Muhammad Iqbal, Muhammad Shahbaz, Owais Bin Qadeer, Kanwal Nazir, Muhammad Naeem, Muhammad Shahzad Afzal, Muhammad Babar Imran

**Affiliations:** Department of Nuclear Medicine, PINUM Cancer Hospital, Jail road Faisalabad, Faisalabad, Pakistan

**Keywords:** COVID-19, Coronavirus, Nuclear medicine, Pandemic, Scanning

## Abstract

**Background:**

With the global surge in COVID-19 pandemic, it has become inevitable for everyone, inclusive of nuclear medicine personnel, to play their role in combating and containing its transmission. During fall 2019, China encountered a novel coronavirus in Wuhan city which was later on termed as COVID-19. The pneumonia caused by COVID-19 is characterized by dry cough, fever, fatigue, and shortness of breathing (dyspnea). Until now, this virus has spread worldwide and continues to cause exponential causalities.

**Main body:**

This global catastrophic scenario calls for stringent measures to control COVID-19 infection. Thus herein, the respective authors have endeavored to review precautionary measures for nuclear medicine department, encompassing its personnel as well as the patients so that intradepartmental transmission can be prevented. This requires development and execution of a robust and dynamic plan elaborating the healthcare guidelines. Hence, our review paper covers the arena of nuclear medicine services in particular.

**Conclusion:**

Nuclear medicine can play its role in mitigating COVID-19 transmission to personnel and patients if provided with ample PPEs and guidelines are strictly followed. With implementing SOPs (standard operating procedures) based on these guidelines, nuclear medicine facilities will be better prepared for impromptu actions in case of any future outbreak while retaining the smooth flow of obligatory healthcare services.

## Background

The widely known virus in the current era, i.e., the novel coronavirus, was first detected in Wuhan city of China in December 2019 and was therefore named as COVID-19. Within a couple of months, it evolved from epidemic into pandemic and was declared as a global health emergency on 11 March 2020 as it had infected 120,000 people with 4373 casualties across 114 countries. The major reason behind this is the quick transmission of this virus from one person to another via respiratory droplets as well as community binge; there could also be a number of asymptomatic infected carriers to contribute to its spread [1]. As per worldometer, 13.2 million patients have been confirmed to be infected with COVID-19, and it has caused 575,000 deaths worldwide so far. In Pakistan, there are 254,000 confirmed COVID-19 cases and 5320 casualties.

**Fig. 1:**
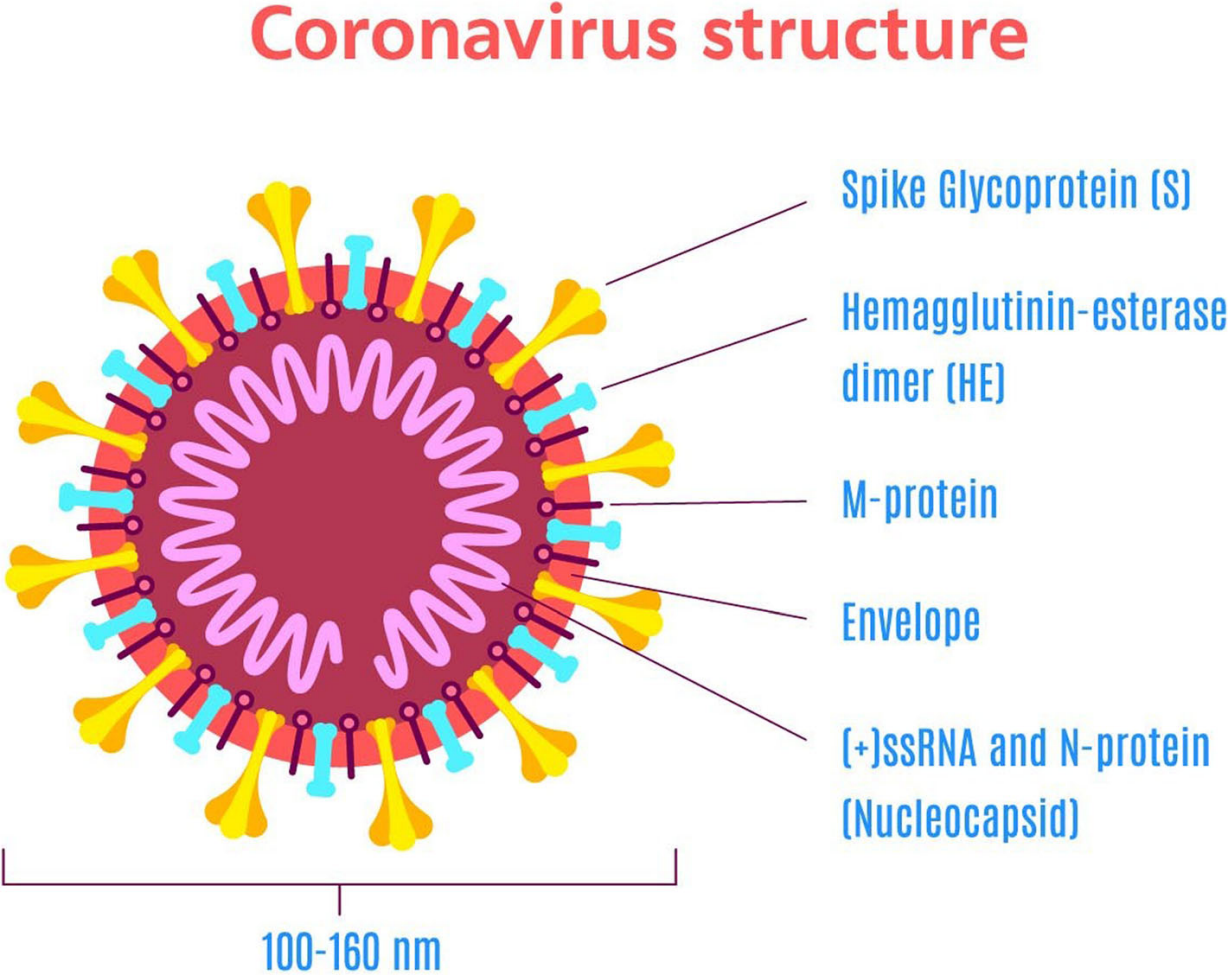
COVID-19 structure [1, 2]

Coronaviruses (CoVs) belong to Coronaviridae family and are enveloped and non-segmented while carrying a single stranded, positive sense ribonucleic acid (RNA). They are very crucial pathogens in terms of infecting humans and other vertebrates such as mouse, bat, snakes, livestock, and birds. They can infect not only the respiratory system but also the hepatic, central nervous, and gastrointestinal system. Previous epidemics have shown the transmission of CoVs from animal to animal and from human to human. The mysterious COVID-19 outbreak in Wuhan has become the research focus worldwide owing to its pandemic state because of its rapid transmission all over the globe. Profound sequencing and investigation performed by several laboratories of China has shown it to be a novel coronavirus by far [2]. As far as its genome and phylogenetic relationships have been studied, it belongs to the Betacoronavirus genera. Its sequence turned out to be greatly similar to syndrome-related coronaviruses (SARS-CoV). It is spherical in shape and is enveloped with pleomorphic envelope (Fig. 1). This envelope encompasses glycoproteins in the form of club-shaped projections. Also, another protein, hem agglutinin esterase (HE), projects from the envelope. However, its detailed pathogenesis still needs to be explored further in order to facilitate the process of its vaccine and drug production [1, 2].

By far, enormous efforts have been done to develop an antiviral agent against CoVs’ entry proteins, polymerases, proteases, and methyl transferases, but none has demonstrated successful clinical trials. Only limited therapeutic results have been obtained using recombinant interferon therapy supported with ribavirin. However, recovered patients’ antibodies and plasma could give scientists a promising dimension to focus on. Additionally, different types of vaccines such as attenuated, inactive, subunit, vector, DNA, or recombinant protein vaccines have also been formulated, and trials have been conducted in animals [3]. All in all, absence of any operative prophylactic or therapeutic approach calls for stringent precautionary measures to control the very transmission as well as reliable prognosis and reporting. As soon as a patient is confirmed to be having the very infection, prompt isolation and supportive treatments should be ensured. All the collected information should also be timely published to spread the awareness and guidance [4].

## Main text

The relatively privileged fact about nuclear medicine is that most of the scans as well as therapies are tackled in outpatient department as follow-up cases whereas new studies are usually dealt in inpatient department after going through COVID-19 screening. On the contrary, an unfortunate fact is that nuclear medicine lacks portable SPECT and PET scanners, and there is constant need to inject the patients with radiopharmaceuticals so the movement of patients cannot be restricted. So it is very crucial for nuclear medical workers to have thorough knowhow of precautionary measures to prevent COVID-19 transmission [1]. A number of articles have been published by far encompassing radiology departments, but guidelines regarding nuclear medicine department are still scarce. In particular, majority of the heart patients who have to go through nuclear cardiology procedures are usually above 60 years of age and also carry other extreme health risks such as diabetes, hypertension, and chronic renal and lung disease. All of these factors make them highly vulnerable for severe conditions developing from COVID-19 [5]. A detailed set of instructions concerning portable scanners, duration of patient contact and scan, and the urgency of management is much needed which has been formulated herein.

Nuclear medicine personnel also fall among the category of first-line healthcare workers who are likely to have exposure of COVID-19. All such facilities should be carrying proper guidelines to deal with suspected or known COVID-19 patients as it is a highly contagious virus. It mainly transmits via respiratory droplets; however, there is a probability of its transmission via getting in contact with a contaminated item or surface (fomite). Thus, all healthcare workers must have a comprehensive understanding of all its potential transmission routes to ensure their and patients’ safety. As far as the respiratory droplets are concerned, they are most likely to spread within 3 ft range but can also move up to 6 ft [6].

On 4 March 2020, the World Health Organization (WHO) recommended to ensure respiratory protection using a standard medical mask in all healthcare provision facilities if no aerosol generation procedure is executed. However, advanced guidelines issued by the Centers for Disease Control and Prevention (CDCP) recommend using N95 or higher filtration mask while working in close proximity with COVID-19 patients or suspects. Personal protective equipment (PPE) has also been recommended to arrange droplet protection which includes a fluid-resistant disposable gown, disposable gloves covering the cuffs of gown, face mask, and eye goggles. Different studies conducted on healthcare providers, who had an exposure to coronavirus, showed that viral transmission can be substantially reduced if contact and droplet diffusion is restricted [7].

**Fig. 2:**
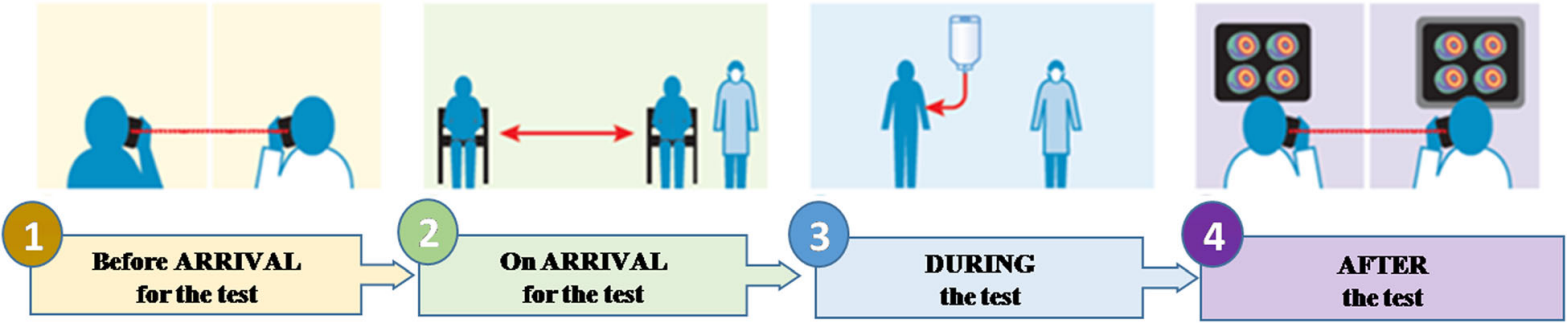
A summary of basic nuclear medicine SOPs against COVID-19 [6]

On the whole, nuclear medicine departments should stay highly alert of COVID-19 suspects and should maintain a compliance with the following WHO’s and regional/institutional precautionary and control measures (Fig. 2):

- Installing hand sanitizer dispensers at all the feasible places of nuclear medicine department and ensuring their use by everyone who passes by. Their regular refilling should also be made certain.
- Various communication tools such as posters and flexes, intranet banners, and virtual staff meeting should be maximally utilized to incline people towards respiratory and sanitary measures.
- Potentially contaminated surfaces such as ultrasound probes, MRI and CT scanning gantries, blood pressure measuring cuffs, all keyboards, and mice must be undergone proper disinfection after each contact with positive or suspected patients. As per international standards, this disinfection can either be achieved by washing with soap/detergent water or by using disinfectants like isopropyl alcohol, ethyl alcohol, iodophor, or sodium hypochlorite.
- An exclusive training of environmental service providers should be conducted so that high-risk surfaces can be professionally cleaned.
- Respective vendors of each equipment should be reached out for the particular and safest disinfectant provision.
- If any of the staff members show slightest symptoms of COVID-19, they should be instructed to stay at home until thorough clinical examination.
- A minimum distance of 3 ft among all individuals must be maintained unless contact is inevitable, such as while injecting or scanning. Nonetheless, PPE must be used.
- Readjusting the scan protocols by choosing the shorter and urgent ones, for example, avoiding multi-day imagining or replacing 2 days myocardial perfusion analysis with 1-day procedure.
- There should be strict policies regarding the visitors. Only one adult (18 years or plus) visitor should be allowed at a time after passing through screening questions and thermal scanning.
- All the training and meetings should be held virtually.
- Personnel should not be traveling unless inevitable, and post-travel observations and measures should be undertaken.
- In order to minimize the intra-transmission risk, patients as well as personnel should be segregated as per the feasibility, i.e., outpatients and corresponding personnel should be kept separated from the inpatients and subsequent personnel. Furthermore, to be safe from a complete shutdown in case of intra-transmission of COVID-19, the staff should be divided into onsite and offsite teams. Onsite team should be dealing with the patients, and offsite should stay away from patients and onsite workers while participating in tele-consultation and online board meetings. There should be a rotation after 14 days, and COVID-19 test should be conducted for the onsite workers before they are sent offsite.
- Lastly, if lung analysis via nuclear medicine scanning shows any COVID-19 symptoms, the suspect and respective visitors and personnel should be promptly informed, quarantined, clinically examined, and treated as required [8–10].

### Step-wise guidelines

**Fig. 3:**
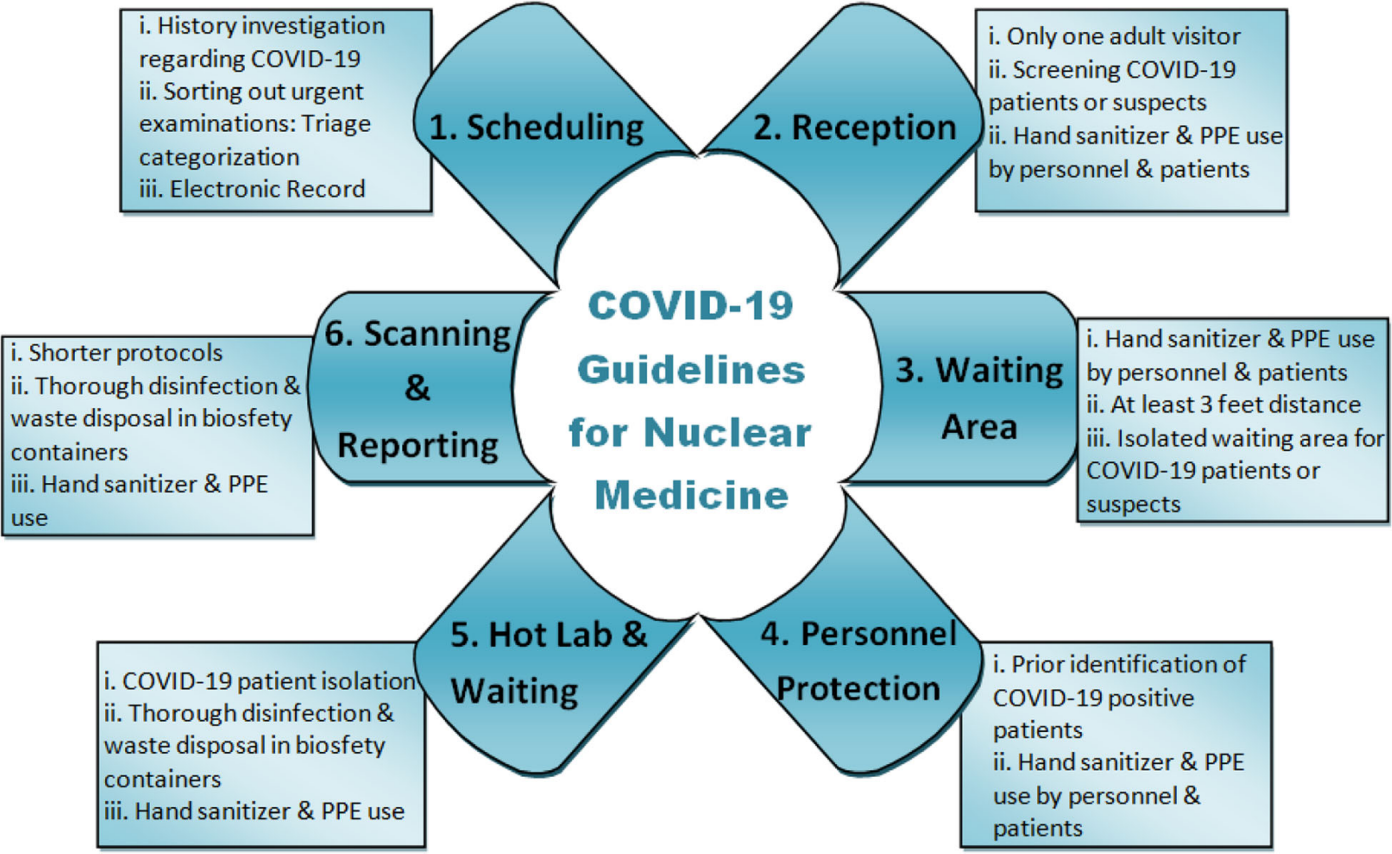
COVID-19 protection and prevention measures in nuclear medicine department [1]

In order to minimize the COVID-19 exposure to healthcare professionals as well as patients, various steps encompassing pre-test, peri-test, and post-test procedures have been listed below (Fig. 3).

#### Pre-arrival instructions

Nuclear medicine team needs to assure the following before patients’ arrival to the premises for examination [11]:

- The risk level associated with the patient in terms of COVID-19 transmission.
- How urgent is this examination, that is, to see if it can be postponed without any health liability. If yes, then for how long it can be postponed.

It is very crucial to contact the patients before their arrival and gather their history regarding COVID-19 risk and compliance with its control measures. Furthermore, any clinical signs or symptoms of COVID-19 should be inquired and noted down such as fever, cough, diarrhea, abnormal fatigue, hyposmia, anosmia, myalgia, ageusia, or dysgeusia. Also, a record of their previous 2 weeks’ traveling or any exposure to COVID-19 patients or high-risk areas should be acquired. Another aspect of these potential risks is the contact with nuclear medicine equipment and respective personnel. If it is feasible for the patients, then, they should be coming to the premises alone, and if it is inevitable to be accompanied, then, history regarding COVID-19 must also be acquired from the accompanying individual. Else, if there is exponential COVID-19 prevalence in a certain area, it may be made necessary to perform COVID-19 testing of outpatients as per some additionally formulated policies [12].

**Fig. 4:**
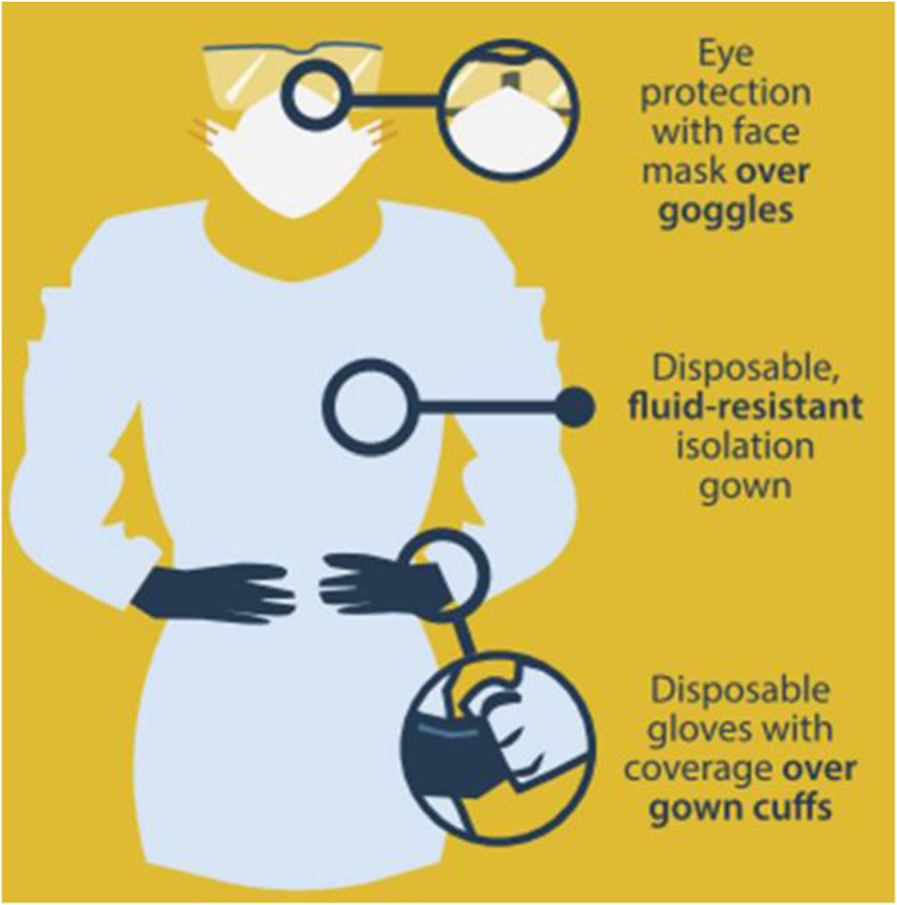
Personal protective equipment for nuclear medicine healthcare providers [4]

In case of calling those patients who have had high-risk exposure to COVID-19 to nuclear medicine facility, stringent cautions should be exercised. However, all healthcare premises are trying their best to restrict the number of out department patients by deferring the patients without an urgent need of examination so that COVID-19 risk can be reduced, and PPE (Fig. 4), sanitization, and sterilization can be conserved owing to their scarcity. There should be such procedures which can potentially help adapt as per any change in the management within a short span of time and can influence subsequent prognosis. On the other hand, such procedures can be postponed which have a relative tendency to identify such sudden modifications and can impact only a long-term prognosis. A detailed judgment at clinical level is necessary to classify these procedures [13].

After gathering pre-arrival patients’ history, they can be divided as per the triage categorization, i.e., priority 1: urgent basis; priority 2: postponed till 2–4 months; and priority 3: postponed till more than 4 months. Maintaining electronic patient history record can help the personnel in deciding the priority category. The patients whose visit is deferred should be provided with a due explanation, and they should be kept under constant follow-up regarding their health status [14].

#### Reception of patients

When the patients arrive to the premises, they should be inquired about any COVID-19 symptoms, especially if they have been to a place where COVID-19 is widespread. The reception desk should be carrying a notice so that patients may self-declare their COVID-19 status. If the nuclear medicine department is large enough to tackle a certain mass of outpatients, rapid thermal scanners may be used for skin temperature screening, like the ones being used at airports. Personnel should keenly observe the patients for any COVID-19 symptoms such as dry cough, fever, and fatigue. Also, it should not be overlooked that there could be asymptomatic COVID-19 carriers among the patients. Therefore, all of them should be strictly made to wear masks. As far as patient isolation is concerned, it should be tried to keep outpatients separate from outpatients. Another considerable fact is that nuclear medicine departments could be accompanying oncology and/or nuclear cardiology patients. So as per the feasibility, immunocompromised patients should be kept separated from other ones and should be dealt by separate personnel [15].

It should be ensured that the waiting area has been provided with wash basin, sanitizer, masks, and tissues to be accessed by the incoming patients. Relevant posters promoting personal hygiene measures should be displayed. There should be enough space (at least 3 ft) among patients to reduce any potential transmission, and the premises should never be overcrowded. Daily-use items at reception like desk, keyboard, and mouse should be regularly disinfected, and the whole reception should be sprayed with disinfectant at the end of the day. PPE must be used by the reception personnel, and they should be sitting behind a plastic or glass screen. There should be a back-up in case if any of the personnel falls sick because of COVID-19 so that continuity of the services can be maintained [16].

#### General waiting area

Similar to the reception zone, general waiting area should also be equipped with easily accessible wash basin, hand sanitizers, masks, and tissue boxes, and patients should be constantly encouraged to utilize these facilities for basic hygiene measures. Likewise, patients should be sitting at least 3 ft away from each other to reduce the transmission risk [17]. If COVID-19 patients have been identified at the reception desk, they should be kept in an isolated waiting area with proper consultation regarding infectious diseases. If they have not gone through COVID-19 test yet, it should be tried to postpone their scans until they get the test done so that post-infection guidelines can be followed. Personnel should make it inevitable for COVID-19 patients to wear masks during all the period of their examination. Any additional instructions formulated by the local authorities must also be put up with [18].

#### Protection for personnel

Frontline personnel of nuclear medicine facility are the most vulnerable to COVID-19 exposure as they will be directly dealing with COVID-19 patients. Therefore, a prior identification of COVID-19 positive patients is inevitable so that the consultant personnel can adopt the due precautions as they will be in physical contact for a considerable span of time with the infected patients. To cope with infected patients, PPE must be used as described before. It may reduce the number of patients who can be examined in a day but it is likely to mitigate the number of positive cases in a particular department [19].

#### Hot lab and post-injection waiting area

Most of the nuclear medicine examinations/scans require injecting a radiotracer whose uptake time may range from several minutes to several hours. There are certain hot labs where these radiotracers are injected into the patients, and then, they are sent to the waiting zones until they are called for the particular scan after the completion of the optimized uptake. Hence, similar precautions/guidelines as suggested for the post-reception waiting area should be adopted here too. Such as all the disinfection and antisepsis techniques should be employed, PPE must be utilized with the due special attention and must be disposed in a biosafety waste container, and hands should be repeatedly sanitized after every procedure [20].

#### Precautions during and after nuclear medicine scanning

**Fig. 5:**
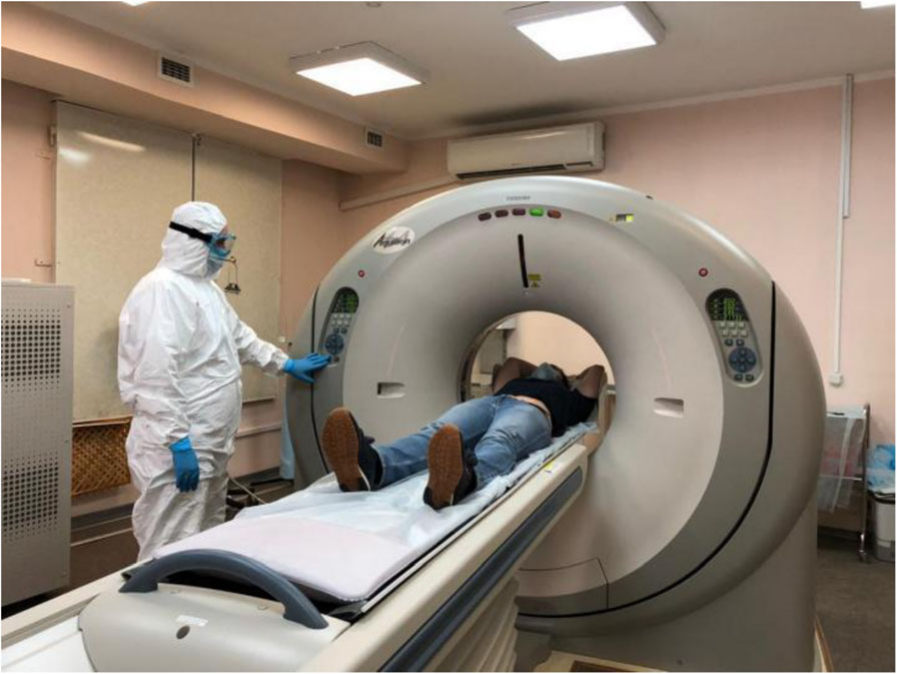
COVID-19 guidelines being followed during nuclear medicine scanning [25]

All the measures suggested by far should be followed during the scanning protocols as well (Fig. 5). A proactive approach should be adopted by the seniors/supervisors by making their staff stay at home in case they are not feeling well. If COVID-19 becomes endemic, bigger nuclear medicine set-ups should try segregating their personnel into at least two categories: one dealing with infected patients and one dealing with non-infected patients so that COVID-19 transmission can be controlled [21]. As pneumonia is one of the complications of COVID-19, a lung scanning may be required. Thus, nuclear medicine facilities should be relatively more vigilant regarding those patients who are advised lung scanning or CT scan of lungs as there could be even asymptomatic carriers among them. As lung complications can be a symptom of various ailments, a potential case should be taken under cautious observation for further clinical scrutiny [22].

As far as scanners are concerned, disposable protective elements should be used, and thorough disinfection (1000 ppm chlorine) should be performed after scanning a COVID-19 positive or potential patient. The whole room should be systematically cleaned and then kept closed for 30–60 min and then terminally cleaned. Then, scans can be continued after a break of 1 h [23]. Additionally, time to time disinfection of all the surfaces and equipment should be performed during the scans, and sheets/linen should be replaced as per the guidelines. Disinfectant fumigation and disinfecting the air conditioning systems may also be incorporated as per the feasibility. In order to expedite the testing process, minimal length protocols should be selected. For acquiring consent, minimum contact of pens and papers should be done, or virtual consent should be obtained prior to the visit so that electronic record can be maintained. Remote image reporting or tele-discussion should be preferred to in-person image reviewing. Also, use of automatic equipment (such as BP cuffs) may be considered [24].

### Unattended aspects

Despite all the above discussed detailed precautionary measures regarding current era of COVID-19 pandemic, there are certain facts that are still unanswered. The following sections deal with these unattended aspects.

#### Necessity of nuclear medicine procedure

We discussed all nuclear medicine procedures in general. However, while adopting a particular approach, an inescapable question arises here. If we have to follow a triage of priority in nuclear medicine procedures, which nuclear medicine procedures must be done without delay based on their emergency need? To answer this, proper categorization based on triage of priority must be carried out to ensure least exposure of nuclear medicine department and personnel.

#### Disinfection of nuclear medicine equipment

Disinfection of nuclear medicine equipment is always an issue, i.e., it is not as simple as disinfecting general surfaces. Such as gamma camera used for nuclear medicine scanning procedures is one of the most expensive medical equipment. It possesses a sodium iodide (NaI) crystal which is hygroscopic and heat sensitive [26]. A thorough review of multiple guidelines for nuclear medicine procedures suggests pursuing vendor guidelines for disinfecting all this expensive equipment. To date, no proper guidelines were available apart from generally recommended disinfectant. If we refer to user manuals, they clearly warn about the drastic effects of humidity and heat on gamma camera crystals. Temperature above 23 °C plays a vital role in sterilization of surfaces but at the same time can be damaging to this expensive medical equipment. Also, direct or indirect effects of these disinfectants on gamma camera crystal when used to disinfect the room are unclear as no instructions are provided from the vendors or warranting authorities.

#### Training of nuclear medicine workers to work in PPE

Working in PPE always remains a challenge and can greatly affect the working efficiency of nuclear medicine workers. Carrying out fine interventions during various procedures can be cumbersome [27]. Therefore, it is critical to provide training for nuclear medicine personnel to work with PPE.

## Conclusion

Although nuclear medicine services have been accentuated for a number of issues owing to present worrisome COVID-19 scenario, it can play its role in mitigating its transmission to personnel and patients if it is provided with ample PPE and guidelines are strictly followed. Once these guidelines have been implemented, nuclear medicine facilities will be better prepared for impromptu actions in case of any future outbreak. Therefore, the purpose of this manuscript is to provide supporting material for nuclear medicine departments so that they may be able to formulate COVID-19 prevention SOPs (standard operating procedures) catalog. Basically, the gist of this whole planning is to place special emphasis on COVID-19 risk reduction to all the involved individuals while retaining the smooth flow of obligatory healthcare service provision. Conclusively, adopting a multifactorial vision encompassing all the steps from pre-arrival patient scheduling to report delivering and strict observation of COVID-19 patients or suspects is the key to achieve the due goals. It must be ensured for each individual to practice hygiene and disinfection measures and utilize required PPE.

## Data Availability

Not applicable

## Abbreviations

CoVs: Coronaviruses
COVID-19: Novel coronavirus 2019
PPE: Personal protective equipment
SOPs: Standard operating procedures
NaI: Sodium iodide
SPECT: Single photon emission computed tomography
PET: Positron emission tomography
CDCP: Centers for Disease Control and Prevention
CT: Computed tomography
MRI: Magnetic resonance imaging
